# A new look at population health through the lenses of cognitive, functional and social disability clustering in eastern DR Congo: a community-based cross-sectional study

**DOI:** 10.1186/s12889-019-6431-z

**Published:** 2019-01-21

**Authors:** Espoir Bwenge Malembaka, Hermès Karemere, Ghislain Bisimwa Balaluka, Anne-Sophie Lambert, Fiston Muneza, Hedwig Deconinck, Jean Macq

**Affiliations:** 1 Ecole Régionale de Santé Publique, ERSP, Faculté de Médecine, Université Catholique de Bukavu, Bukavu, Democratic Republic of Congo; 2 Institute of Health and Society, IRSS, Université Catholique de Louvain, Brussels, Belgium; 3 Department of Epidemiology and Biostatics, School of Public Health, College of Health Sciences, Makerere University, Kampala, Uganda

**Keywords:** Health clustering, WHODAS, Medico-psychosocial, Disability, Community, Eastern DR Congo

## Abstract

**Background:**

The importance of viewing health from a broader perspective than the mere presence or absence of disease is critical at primary healthcare level. However, there is scanty evidence-based stratification of population health using other criteria than morbidity-related indicators in developing countries. We propose a novel stratification of population health based on cognitive, functional and social disability and its covariates at primary healthcare level in DR Congo.

**Method:**

We conducted a community-based cross-sectional study in adults with diabetes or hypertension, mother-infant pairs with child malnutrition, their informal caregivers and randomly selected neighbours in rural and sub-urban health zones in South-Kivu Province, DR Congo. We used the WHO Disability Assessment Schedule 2.0 (WHODAS) to measure functional, cognitive and social disability. The study outcome was health status clustering derived from a principal component analysis with hierarchical clustering around the WHODAS domains scores. We calculated adjusted odds ratios (AOR) using mixed-effects ordinal logistic regression.

**Results:**

Of the 1609 respondents, 1266 had WHODAS data and an average age of 48.3 (SD: 18.7) years. Three hierarchical clusters were identified: 9.2% of the respondents were in cluster 3 of high dependency, 21.1% in cluster 2 of moderate dependency and 69.7% in cluster 1 of minor dependency. Associated factors with higher disability clustering were being a patient compared to being a neighbour (AOR: 3.44; 95% CI: 1.93–6.15), residency in rural Walungu health zone compared to semi-urban Bagira health zone (4.67; 2.07–10.58), female (2.1; 1.25–2.94), older (1.05; 1.04–1.07), poorest (2.60; 1.22–5.56), having had an acute illness 30 days prior to the interview (2.11; 1.24–3.58), and presenting with either diabetes or hypertension (2.73; 1.64–4.53) or both (6.37; 2.67–15.17). Factors associated with lower disability clustering were being informally employed (0.36; 0.17–0.78) or a petty trader/farmer (0.44; 0.22–0.85).

**Conclusion:**

Health clustering derived from WHODAS domains has the potential to suitably classify individuals based on the level of health needs and dependency. It may be a powerful lever for targeting appropriate healthcare service provision and setting priorities based on vulnerability rather than solely presence of disease.

**Electronic supplementary material:**

The online version of this article (10.1186/s12889-019-6431-z) contains supplementary material, which is available to authorized users.

## Background

The importance of viewing health from the standpoint of functional, cognitive and social disability dimensions is critical at primary health care level. It is no longer debatable that health is a dimension inextricably interwoven with all other aspects of life, daily life, working life, family life, and community life [1]. Health is increasingly considered as a human capital resource and a whole, personal, situation-specific phenomenon [2], rather than the absence of disease [3–5]. Despite such a consensus, primary care activities are still largely structured around diseases control and mortality of sub-populations rather than promoting comprehensive person-centered care [6].

When one addresses a person’s health or community health, the life situation as a whole must be put into perspective and strategies for improving health needs must be grounded on factors conducive to good quality of life. Therefore, prioritising (community) care through population stratification based on functional, cognitive and social disability dimensions may be useful for comprehensiveness and quality of service provision. This has not yet been sufficiently explored in low- and middle-income countries (LMICs). Few studies from both high-income countries and LMICs examined these health dimensions of sub-populations, but mostly in the elderly or had a limited focus on hospital- and disease-based outcomes [7].

The literature is insistently advocating the necessity to broaden the perspective of ‘health measurement’ by looking at functional and social status as part of individual and community health [8, 9]. With recognition that health complexity encompasses and transcends the mere concept of physical morbidity, good health can be viewed as the ability to adapt and to self-manage, with emphasis put on social and personal resources as well as physical capacity [10]. In the era of steadily rising high burden of chronic comorbidities, new health considerations such as functional dependence, cognitive functioning, disability and frailty are becoming of greater importance. But little is known on how these innovating insights into individuals and community health can be leveraged so that to ensure appropriate health services to people most in need and pave the way to universal health coverage and progress towards the health sustainable development goals (SDGs).

The World Health Organization (WHO), echoing the need for a holistic approach to assessing health both at individual and community levels, developed appropriate tools that allow a better understanding and estimation of the impact of any health condition in term of functioning. The International Classification of Functioning, Disability and Health (ICF) published by the WHO in 2001 has proved to be a useful and valid framework integrating function and disability with health conditions and contextual factors [11]. Besides, the WHO Disability Assessment Schedule 2.0 (WHODAS), drawing essentially on the ICF framework, provides a standardized approach to measuring health and disability across cultures [12].

Primary health care services in sub-Saharan Africa and other low- and middle-income, particularly in post-war settings, are often considerably configured to donors-dictated disease-based indicators [13]. To some extent, this furtively leads to denaturalisation of the fundamental goal of primary health care. In such situations, individual and community health are confined to the narrow physical or biological aspects of health, ignoring the broader dimensions of health that are useful for a person’s life. There is dearth of context-specific data on how to identify vulnerability-based sub-populations of complex patients who may benefit from targeted care management strategies in resources-constrained settings.

The aim of this study was twofold. First, we propose a new stratification of population health in a sample of adults with diabetes or hypertension, mothers of children with acute malnutrition as well as their respective informal caregivers and neighbours from the standpoint of functional, cognitive and social disabilities. Second, we identified covariates of population health in rural and semi-urban eastern DR Congo settings. Our overall goal is to better inform healthcare strategies and improve health services organisation in rural and/or post-war settings.

## Methods

We conducted a community-based cross-sectional survey in adults with self-reported diabetes and/or hypertension, mother-infant pairs with severe acute malnutrition, and their informal caregivers and neighbours between December 2017 and March 2018. These sub-population categories were deliberately selected to help gain a better insight into diverse perspectives of health status patterns observable at primary healthcare level in South Kivu. In addition, we deemed high the likelihood of complex medico-psychosocial conditions among individuals presenting with these three ‘tracer’ conditions [14, 15]. Finally, these health problems are prevailing in South Kivu and are relatively easy to identify even at primary care level in resources-constrained settings. Indeed, the prevalence of acute malnutrition in South-Kivu is one of the highest in the world with up to 8% of children younger than 5 years being wasted [16]. In 2011, the estimated prevalence of diabetes in this region was 4.9% in urban areas and 3.2% in rural areas while hypertension was found in 41.4 and 38.1% of urban and rural residents respectively [17].

### Study settings

South-Kivu is an Eastern DR Congo province as large as 65,103 km^2^, lying in the Great Lakes region of Africa. This province shares land borders with Burundi and Rwanda and borders the provinces of North-Kivu, Maniema and Katanga. The Kivu region has been a theatre of civil and political unrest over the last two decades, resulting in socio-economic instability, destruction of societal structures and, to a significant extent, dysfunction of the health system. With an estimated population of 6,932,107 inhabitants in 2012 [18], South-Kivu is a predominantly rural province with nearly 70% of the population living in rural areas. The operational unit and primary care level of the health system in DR Congo is the health center with the health area as catchment unit.

### Sampling procedure and selection of participants

A multi-stage sampling approach was used. Six health areas (Bideka, Burhale, Kabushwa, Lumu, Lwiro and Nyamuhinga) spanning four health zones (Bagira-Kasha, Katana, Miti-Murhesa and Walungu) were selected because of their large catchment area, geographical accessibility, experience and quality of records keeping. A strong network of over 240 community health workers (CHWs) operated in these health areas covering over 100,000 inhabitants. Given the logistic, geographical, time and–to a lesser extent–security constraints, we purposively confined the sampling to villages nearest to the health centre. This was also partly because this study was part of a broader and longer-term research for development project that set to find out whether changes in the way health services are provided (by focusing on psycho-medico-social status additionally to the disease) at health centres in rural and post-war African contexts would change the health status of a population.

We initially aimed to recruit at least 90 patients (with any of the three conditions aforementioned) and an equal number of informal caregivers and neighbours in each of the six selected health areas. Within each health area, villages nearest to the health centre were selected. At village level, CHWs were recruited. Studies have shown that, with a minimum training, CHWs can effectively participate in screening, health promotion interventions and management of malnutrition [19], diabetes or hypertension [20, 21]. They benefited from a half-day refreshment training on the community diagnosis of severe acute malnutrition based on mid-upper arm circumference measurement (MUAC) equal or below (≤) 11.5 cm and/or presence of nutritional oedema. The refreshment training was deemed necessary to ensure a correct identification of mother-infant pairs with child acute malnutrition given that untrained mothers or caretakers are unlikely to properly detect and self-report acute malnutrition in their children [22]. CHWs were also assigned to identify households in which adults with self-reported diabetes or hypertension lived. During the data collection phase, the data collection team was introduced to each household in which a person of interest was identified. The purpose of the study was explained to the head of the household and permission to carry out the interview was asked. People with diabetes and hypertension were selected if being diagnosed for at least 6 months. Mothers were selected if being a mother of a child presenting with severe acute malnutrition. If the targeted person was absent, the data collectors could proceed to the next targeted household on the list and come back the following day until the person was found. A written and signed consent to participate in the study was sought before the interview started in the same household, an informal caregiver was identified and asked to consent to the study. For caregivers below 18 years of age, the consent was required from a parent or guardian. For every household in which a patient was recruited, a community member in the nearest neighbourhood was randomly selected by spinning a pen and following the direction in which it pointed. At this stage, an adult with the closest age and ideally (but not always) with the same sex as the neighbour patient was approached and asked to participate in the study, after providing a written consent. If in the selected neighbouring household there was no consenting adult, interviewers could move to a next household chosen through the same random process until they found a consenting adult. At the end, all participants had to be residents of the health area for at least 6 months and at least 15 years of age. People who refused to provide an informed consent or were severely ill, physically or mentally unable to withstand an interview were excluded.

### Data collection and instruments

A simple identification form was used by the CHWs during the phase of identifying households in which patients with known morbidity lived, within the entire health area. This helped us generate a sampling frame with information on age, sex, village of residence and type of morbidity. A structured and pre-tested paper-based questionnaire designed to capture socio-demographic and health characteristics data was administered to a convenience sample all identified individuals living in villages nearest to health centres, their informal caregivers and randomly selected neighbours by trained research assistants who were all nurses.

To assess the functional and social disability related to health condition, we used the WHO Disability Assessment Schedule 2.0 (WHODAS). WHODAS is a multidimensional and cross-cultural questionnaire with 36 items assessing an individual’s cognition, mobility, self-care, getting along with people, life activities and participation in society. It is short to administer (about 20 min) both at clinical and community levels and across all diseases. WHODAS has been validated and frequently used in LMICS [23, 24], with a high internal consistency (Cronbach’s alpha) ranging from 0.77 in South-African women [25] to between 0.82 and 0.98 in people with severe mental disorders and their caregivers in rural Ethiopia. It also is able to detect small changes over time [26]. In addition, the WHODAS-child adapted from the adult WHODAS 2.0 has shown an 84% internal consistency with high test-retest and inter-rater reliability (r  =  0.83 and intraclass correlation coefficient   =  0.88) in Rwandan children [27].

The WHODAS was translated to Kiswahili (national language spoken in eastern DR Congo) according to a rigorous translation protocol to ensure cross-cultural and conceptual equivalence. One French-speaking translator from the school of languages of the Université Catholique de Bukavu and whose mother tongue is Kiswahili carried out the translation. A bilingual panel comprised of the principal investigator, key health professionals working in the health areas of study and community health workers leaders reviewed the translated version in order to address its potential cross-cultural inadequacies in terms of incomprehensibility or lack of clarity.

### Variables and measurement

WHO developed a conceptual framework for action on the social determinants of health [28], which we found complementary to that developed earlier on by LF Berkman, T Glass, I Brissette and TE Seeman [29]. Drawing on both frameworks, we examined social (including social cohesion), demographic and economic status as possible explanatory parameters. Socio-demographic characteristics included among other variables age (measured on a continuous scale in completed years), gender (male or female), education (continuous variable measured as complete years of schooling) or household size (number of people sleeping in the same house and eating from the same cooking pot) or health zone of residence. Some categorical variables needed to be recoded to obtain sufficient numbers in strata for ease of the comparisons. This was, for example, the case for marital status, tribe or occupation. Social cohesion and networking were approximated by regularly attending church activities and being member of a local socio-economic or savings network. To define the socio-economic status, we ran a Multiple Correspondence Analysis on household assets and housing characteristics to create wealth indices [30] based on ownership of a television, a radio, a computer, a manufactured bed, small animals, cattle, land, a bicycle, a motorcycle and on housing characteristics including pavement and permanent, semi-permanent or temporary structure. We then derived five socio-economic quintiles from wealth indices. The two lowest (poorest 40%) and the two middle (40%) quintiles were respectively merged following an approach suggested by D Filmer and LH Pritchett [31]. We ended up with three socio-economic classes (least poor, middle poor, poorest).

The main dependant variable under study was functional and social disability defined as a three-level ordinal variable resulting from a Principal Component Analysis (PCA) with clustering performed on the six WHODAS domains scores (see explanation here below).

### Data management and analyses

Data were entered in EpiInfo7 and exported to Stata 15 for exploratory analyses. We used a three-stage WHODAS scoring strategy based on the complex and Item Response Theory (IRT) scoring algorithm. We first added up the recoded item scores within each domain. All six domains scores were totaled prior to converting the summary score into a metric ranging from 0 to 100 (where 0 = no disability; 100 = full disability) (Üstün et al., 2010). This algorithm was implemented in Stata 15.

The distribution of continuous variables was assessed graphically and statistically using the Shapiro-Wilk test. Extreme and implausible outlying values were checked for and set to missing. Qualitative variables were summarized in frequencies and proportions while continuous variables were described in terms of mean with standard deviation (SD) or median with interquartile range (IQR) depending on the shape of the distribution.

To define medico-psychosocial clusters, we first ran a principal component analysis on seven summary scores of the WHODAS domains. We then performed a hierarchical clustering of the principal components based on Ward’s method and using the FactoMineR software package in R [32]. Three ordered clusters were created and termed cluster 1, cluster 2 and cluster 3. We used chi-squared and Kruskal-Wallis tests to compare the characteristics of the study participants by enrolment status or clustering.

To establish the factors associated with functional and social disability clustering, we did the inter-cluster comparison using a mixed-effects ordinal (proportional odds) logit regression model with cluster as a fixed effect and health area as a random effect. This strategy enabled us to take into account the inherent non-independence of socio-demographic factors at health area level, thus ensuring more accurate standards errors for the measures of association between within-health area characteristics and disability clusters. The proportional odds model was favoured over the other ordinal models since the former is most suited to studies under which the outcome is obtained from categorizing a certain underlying continuum. In addition to its greater statistical power to detect differences in a relatively smaller sample [33], this model often generates much simpler interpretable coefficients, even when the order of the outcome is reversed (in which case only the sign of the coefficient is changed) [34]. We used a backward elimination strategy to build the regression model, guided by Wald’s tests and the principle of parsimony. Variables were hierarchically selected into the multivariable model in three stages, based either on a *p*-value equal to or below 0.2 or on public health plausibility as suggested by CG Victora, SR Huttly, SC Fuchs and M Olinto [35]. Socio-demographic factors were selected first. We then included household attributes before adding proximate factors reflecting physical health impairment. Multicollinearity between explanatory variables was assessed using the Variance Inflation Factor (VIF). A VIF greater than 4 was suspected of collinearity. We reported Crude Odds Ratios (COR) and adjusted odds ratios (AOR) with their 95% confidence intervals and *p* values. We regarded a type one error (α) < 5% as statistically significant. We used R 3.3.5 and Stata 15 software for the analyses.

### Ethical considerations

Respondents provided singed informed consent for participation in the study, either by written signature or by fingerprints, depending on literacy. Child assent was obtained for respondents below 18 years of age, after a parent or guardian’s consent. Ethical approval for the study was obtained from the Université catholique de Bukavu Ethics Committee and the *Hospital*-Faculty *Ethics Committee* of UC Louvain.

## Results

### Background characteristics of the study population

Of the 1609 participants approached by data collectors in the field, 1266 provided valid information on functional and social disability. The general background characteristics of the study sample are presented in Table 1. The majority of the participants were female (63.6%), belonging to the indigenous Shi tribe (91.1%) and married (68.8%). The mean (SD) age was 48.3 (18.7) years. Participants lived in bigger size households [median (IQR): 6.5 (5–9)] compared to the national median of 5.3. Farming or petty trading were the main occupation for over half of the heads of households (55.8%). While 62.5% of the respondents claimed to be catholic, about one quarter (27.2%) reported to be members of any church organization with over half (52.4%) of all respondents attending church at least once a week. The median (IQR) duration of schooling was 6 (3–10) years. Nearly six in ten respondents (57%) did not listen to radio even once a week and less than 19.3% reported being members of local saving cooperatives.

**Table 1:** General characteristics of the study population

Variable	Descriptive statistics
Health zone	
Bagira	523 (34.1)
Katana and Miti-Murhesa	502 (32.7)
Walungu	509 (33.2)
Gender	
Female	1003 (63.6)
Male	573 (36.4)
Age	48.3 (18.7)
Socio-economic status	
Least poor	325 (20.3)
Middle	659 (41.2)
Poor	615 (38.5)
Occupation of head of household	
Formal worker	155 (10.7)
Informal worker	189 (13.0)
Farmer or petty trading	810 (55.8)
No profession	298 (20.5)
Participant	
Patient	450 (32)
Informal caregiver	492 (35)
Neighbor	463 (33)
Tribe	
Shi	1446 (91.1)
Others	142 (8.9)
Marital status	
Never married	168 (10.8)
Married	1072 (68.8)
Divorced/widowed/separated	322 (20.4)
Religion	
Catholic	986 (62.5)
Protestant	531 (33.6)
Muslim and others	62 (3.9)
Church membership	
Yes	415 (27.2)
No	1113 (72.8)
Church attendance frequency	
< = Once a week	792 (52.4)
2–3 times a week	462 (30.5)
> = 4 times a week	259 (17.1)
Education (years)	6 (3–10)
Household size	6.5 (5–9)
Preschool aged children in household (*n* = 1011)	1 (0–2)
School-aged children in household	2 (1–3)
Adults in household	3 (2–4)
Saving organization membership	
Yes	306 (19.3)
No	1275 (80.7)
Weekly frequency of listening to radio	
Less than once a week	584 (57)
At least once a week	440 (43)

Data are n (%), mean (SD) and median (IQR)

### Proposed clustering of the study population from the perspective of functional and social disability

**Fig. 1:**
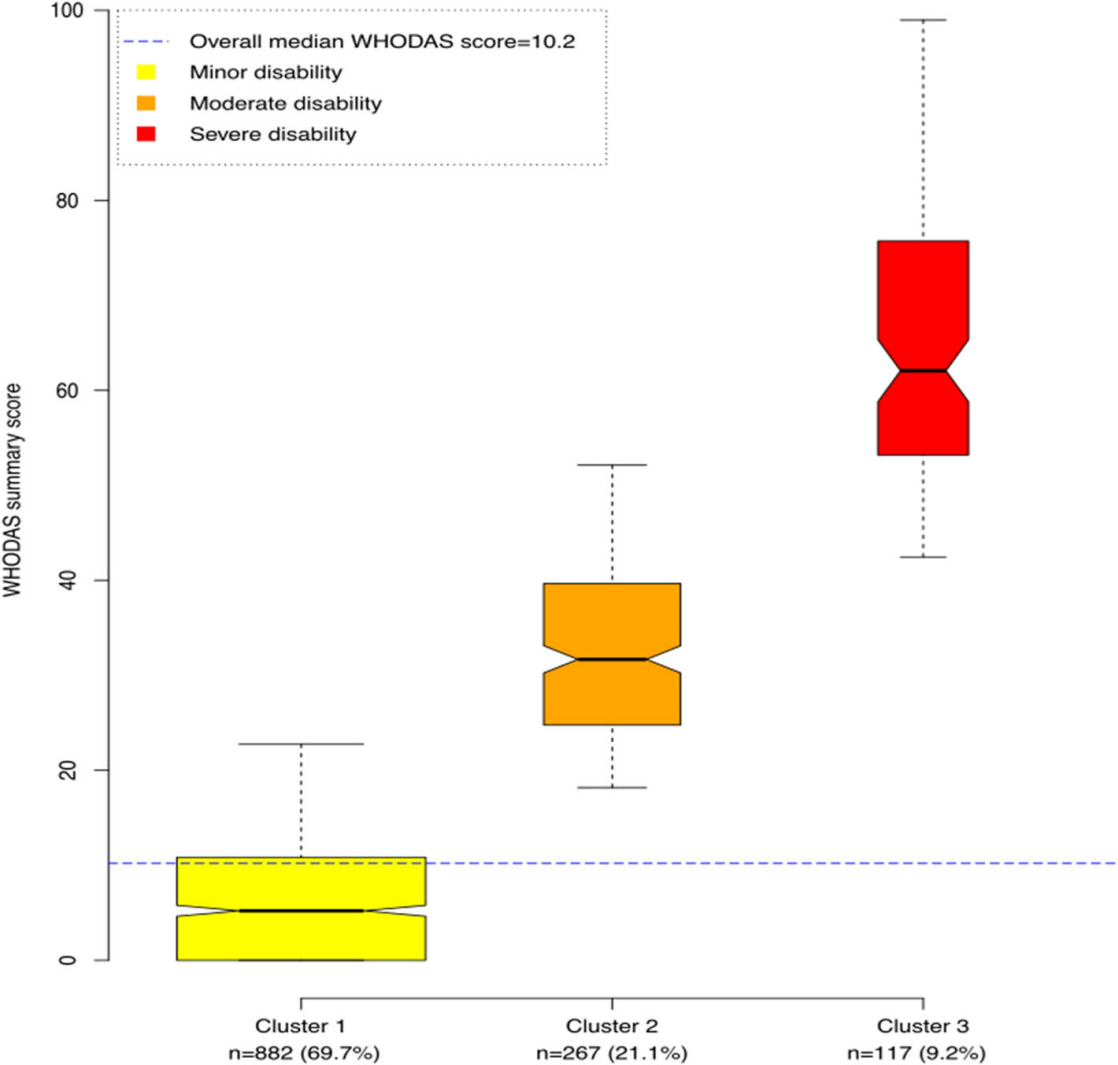
Health status of the study population from a functional and social disability perspective

The hierarchical clustering of the principal components of seven WHODAS domains scores resulted in three ordered categories of functional, cognitive and social disabilities termed cluster 1, cluster 2 and cluster 3 (Fig. 1).

The characteristics of the study population by cluster are displayed in Table 2. Of the 1226 respondents with valid WHODAS data, over two-thirds (69.7%) were found in cluster 1, with a median (IQR) WHODAS score [5.2 (0–10.9)] below that of the whole study population. Cluster 2 had 21.1% of the respondents with a median (IQR) WHODAS score of 31.7 (24.7–39.7). Respondents in cluster 3 (9.2%) had the poorest health status from the functional, cognitive and social disability standpoint with median (IQR) WHODAS score of 62.1 (53.2–75.7) (Table 3). The trend was consistent among all the WHODAS domains; the higher the cluster order, the more worrying the health status of the individuals. Half of the study population had a summary WHODAS score below 10.2 (Additional file 1).

**Table 2:** Distribution of the study population by clusters of functional and social disability representing the levels of complex medico-psychosocial problems

Variable	Cluster 1	Cluster 2	Cluster 3	*P* value
WHODAS and morbidity factors				
Overall WHODAS	5.2 (0–10.9)	31.7 (24.7–39.7)	62.1 (53.2–75.7)	0.001
Communication and understanding	0 (0–12.5)	41.7 (25–54.2)	70.8 (54.2–91.7]	0.001
Mobility	0 (0–5.0)	30.0 (20.0–45.0)	65.0 (50.0–80.0)	0.001
Self-care	0 (0–0)	0 (0–12.5)	50.0 (31.2–75.0)	0.001
Getting along with people	0 (0–15.0)	25.0 (15.0–35.0)	55.0 (30.0–-70.0)	0.001
Household activities	0 (0–12.5)	43.7 (25.0–56.2)	75.0 (62.5–93.7)	0.001
Work or school activities	0 (0–12.5)	43.7 (31.2–56.2)	81.2 (68.7–93.7)	0.001
Social participation	0 (0–16.6)	40.6 (28.1–53.1)	71.9 (59.4–84.4)	0.001
Enrolment status of the participant				< 0.001
Patient	148 (18.7)	133 (58.3)	78 (76.5)	
Caregiver	333 (42.2)	44 (19.3)	13 (12.8)	
Neighbor	309 (39.1)	51 (22.4)	11 (10.8)	
Acute illness/30 days				< 0.001
No	599 (67.9)	113 (43.3)	21 (18)	
Yes	283 (32.1)	154 (57.7)	96 (82)	
Child malnutrition				0.417
No	843 (95.6)	259 (97)	114 (94.4)	
Yes	39 (4.4)	8 (3)	3 (2.6)	
Blood pressure				< 0.001
Normal	543 (68.6)	115 (44.6)	32 (27.8)	
Self-reported HT	147 (18.6)	120 (46.5)	72 (62.6)	
Fortuitous HT discovery	101 (12.8)	23 (8.9)	11 (9.6)	
Diabetes				< 0.001
No	758 (94.7)	192 (80.3)	87 (81.3)	
Yes	42 (5.3)	47 (16.7)	20 (18.7)	
Socio-demographic characteristics				
Place of residence				< 0.001
Urban	329 (39)	67 (26.2)	28 (24.6)	
Rural	514 (61)	189 (73.8)	86 (75.4)	
Age	42.9 (16.4)	58.2 (15.3)	63.0 (18.0)	< 0.001
Sex				0.001
Female	519 (59.9)	187 (70.6)	82 (71.3)	
Male	348 (40.1)	78 (29.4)	33 (28.7)	
SES				0.107
Least poor	199 (22.6)	49 (18.5)	19 (16.2)	
Middle	361 (40.9)	102 (38.5)	44 (37.6)	
Poorest	322 (36.5)	114 (43)	54 (46.2)	
Health zone				< 0.001
Bagira	329 (39.0)	67 (26.2)	28 (24.6)	
Miti-Katana	324 (38.4)	67 (26.2)	48 (42.1)	
Walungu	190 (22.6)	122 (47.6)	38 (33.3)	
Marital status				< 0.001
Never married	116 (13.4)	3 (1.2)	2 (1.8)	
Married	628 (72.4)	192 (73.8)	64 (57.1)	
Divorced/widowed/separated	123 (14.2)	65 (25)	46 (41.1)	
Tribe				0.073
Shi	782 (89.4)	249 (93.6)	108 (93.10)	
Others	93 (10.6)	17 (6.4)	8 (6.9)	
Religion				0.254
Catholic	524 (59.4)	96 (36)	41 (35)	
Others	358 (40.6)	171 (64)	76 (65)	
Listening to radio				< 0.001
No	346 (39.2)	80 (30)	32 (27.4)	
At least once a week	536 (60.8)	187 (70)	85 (72.7)	
Occupation				0.004
Formal salaried	85 (10.4)	19 (7.8)	15 (14.4)	
Informal	213 (25.9)	26 (10.7))	13 (12.5)	
Farmer/petty trading	359 (43.7)	147 (60.2)	42 (40.4)	
No profession	164 (20)	52 (21.3)	34 (32.7)	
Saving organization membership				0.013
No	699 (80)	225 (85.2)	84 (72.4)	
Yes	175 (20)	39 (14.8)	32 (27.6)	
Church attendance/week				0.170
< = 1 time	452 (54.1)	125 (48.6)	56 (51.4)	
2–3 times	235 (28.2)	93 (36.2)	32 (29.6)	
> =times	148 (17.7)	39 (15.2)	21 (19.3)	

*SES* socio-economic status, *HT* hypertension

**Table 3:** Unadjusted and multivariable analysis of the determinants of clustering with regard to social and functional disability

Variable	COR (95% CI)	P value	AOR (95% CI)	*P* value
Status of the participant				
Neighbours	1 (reference)		1(reference)	
Caregivers	0.68 (0.58–1.31)	0.497	0.63 (0.37–1.09)	0.098
Patients	9.56 (6.62–12.86)	< 0.001	3.44 (1.92–6.15)	< 0.001
Health zone				
Bagira	1 (reference)		1 (reference)	
Miti-Murhesa and Katana	1.20 (0.579–2.50)	0.114	1.90 (0.82–4.41)	0.134
Walungu	2.79 (1.35–5.76)	< 0.006	4.67 (2.07–10.58)	< 0.001
Sex				
Male	1 (reference)		1 (reference)	
Female	1.91 (1.33–2.30)	< 0.001	2.1 (1.25–2.94)	0.003
Age	1.07 (1.06–1.08)	< 0.001	1.05 (1.04–1.07)	< 0.001
Household size	0.94 (0.9–0.98)	0.005	1.01 (0.95–1.08)	0.760
Living in conjugal union				
Yes	1 (reference)		1 (reference)	
No	0.63 (0.48–0.84)	0.001	1.37 (0.80–2.35)	0.760
Education of head of household	0.91 (0.88–0.94)	< 0.001	0.97 (0.92–1.02)	0.213
Occupation head				
Employed	1 (reference)		1 (reference)	
Informal work	0.48 (0.27–0.84)	0.010	0.36 (0.17–0.78)	0.010
Petty trading/farming	1.01 (0.63–1.61)	0.974	0.45 (0.22–0.85)	0.016
No occupation	1.91 (1.12–3.28)	0.018	1.01 (0.45–2.29)	0.975
SES				
Least poor	1 (reference)			
Middle	0.87 (0.60–1.26)	0.455	1.67 (0.93–3.02)	0.088
Poorest	1.28 (0.88–1.86)	0.200	2.60 (1.22–5.56)	0.014
Listening to radio				
No	1 (reference)		1 (reference)	
Yes	0.88 (0.68–1.42)	0.342	1.49 (0.91–2.45)	0.113
Acute illness during past 30 days				
No	1 (reference)		1 (reference)	
Yes	5.08 (3.81–6.78)	< 0.001	2.11 (1.24–3.58)	0.006
Child severe acute malnutrition				
No	1 (reference)		1 (reference)	
Yes	0.81 (0.40–1.62)	0.549	0.84 (0.30–2.37)	0.739
Chronic morbidity				
Absent	1 (reference)		1 (reference)	
Diabetes or hypertension	7.66 (5.58–10.52)	< 0.001	2.73 (1.64–4.53)	< 0.001
Diabetes and hypertension	15.09 (7.85–29.02)	< 0.001	6.37 (2.67–15.17)	< 0.001

*COR* crude odds ratio, *AOR* adjusted odds ratio

The age of the respondents and the proportion of women increased with cluster ordering. The majority of respondents in cluster 3 were female (71.3%) and on average 63.0 (18.0 SD) years old and likely to be older than those in lower clusters (*p* < 0.001). The clustering was independent on the socio-economic status (*p* = 0.107) but dependent on the place of residence (p < 0.001). In fact, it was more likely to find participants in cluster 3 in rural areas (Miti-Murhesa, Katana and Walungu health zones) than in semi-urban areas (*p* < 0.001).

Clustering depended on the marital status of the respondents (*p* < 0.001). Only 1.8% of the respondents in cluster 3 never married, 57% were married and 41.1% either were divorced, separated or widowed. Clustering was also dependent on hypertension status, diabetes status and history of acute illness in the 30 days prior to the interview. Over two-thirds of the respondents in cluster 1 (68%) had a normal blood pressure, against 44.6% in cluster 2 and 27.8% in cluster 3 which had 62.6% of its constituents presenting with self-reported hypertension (Table 3). Diabetes was more common in cluster 3 (18.7%) than in other clusters (p < 0.001). Four in five people in cluster 3 reported an acute illness in the 30 days prior to the interview against 32.1% in cluster 1. Clustering was independent of acute malnutrition status of the child, tribe, religion and church attendance, but dependent on occupation. It was more likely to find individuals without profession in cluster 3 compared to cluster 2 and cluster 1 (*p* = 0.004) and respondents in 3 were more likely to be members of local saving or development cooperatives than those in cluster 2 and cluster 1 (*p* = 0.013). Listening to radio at least once a week, a proxy for access to information, was likely to be more frequent in cluster 3 relative to cluster 2 and cluster 1 (*p* < 0.001).

### Covariates of disability-based health status clustering

The crude and adjusted odds ratios of health status clustering based on functional, cognitive and social disability are presented in Table 3. The factors associated with clustering were being a patient compared to a neighbour (AOR: 4.67; 95% CI: 2.07–10.58), being a resident of rural Walungu health zone to semi-urban Bagira (AOR: 4.67; 95% CI: 2.07–10.58), being female (AOR: 2.1; 95% CI: 1.25–2.94), aging (AOR: 1.05; 95% CI: 1.04–1.07), doing informal work compared with being employed (AOR: 0.36; 95% CI: 0.17–0.78), being petty trader or farmer relative to being employed (AOR: 0.45; 95% CI: 0.22–0.85), being poorest compared to being least poor (AOR: 2.60; 95% CI: 1.22–5.56), acute illness 30 days prior to the interview (AOR:2.11; 95% CI: 1.24–3.58), and presenting with either diabetes or high blood pressure (AOR: 2.73; 95% CI:1.64–4.53) or both (AOR: 6.37; 95% CI: 2.67–15.17) to not presenting with either condition.

## Discussion

This community-based study proposes a new way of stratifying population health in function of dependency or disability and social context rather than in function of specific diseases. Similar approaches have been quite frequently studied in high-income countries but scantily tested in LMICs. The implied hypothesis is that this way of stratifying population health may be a powerful lever for change in healthcare prioritization processes.

### A three-layered stratification strategy focusing on functionalities and leading to new strategies

The pyramidal distribution of the study population in three clusters with 9.2% participants with higher disability scores (cluster 3) is different from the few available studies using similar grouping approaches, which nearly all come from high-income countries. SI Vuik, E Mayer and A Darzi [36] classified patients based on healthcare utilization in England and identified 22% of the participants as patients with high health needs. A household-based survey conducted in France by T Lefevre, C Rondet, I Parizot and P Chauvin [37] found that 30% of the study participants were in the cluster of largest primary care users, which may correspond to cluster 3 in our analyses. The observed differences in the proportion of individuals in high healthcare needs clusters between our findings and those from high-income settings can partly be due to the heterogeneity in study design and outcome measurements; therefore, the comparison with our study can only be indirect. Both studies based their outcome measurements on health service utilization. Moreover, the former study used hospital data that may represent people with lower access to healthcare services or with tacit non-disease based healthcare needs, such as social support of social participation. Additionally, a higher life expectancy and aging of the population in high-income countries could explain the higher proportion of individuals with more healthcare needs in these studies compared to our study.

In our study sample, the participants in cluster 3 (117 or 9.2%) would need particular healthcare attention compared to those with middle health and disability concerns in cluster 2 (267 or 21.1%) or those with minor health and disability concerns in cluster 1 (882 or 69.7%). Furthermore, by changing the prioritization process, not all diseased people need the same level of support. For example, 18.7% (148) of the participants in cluster 1 were living with diabetes or hypertension, or were mothers with an acutely malnourished child, while 23.5% (24) of the respondents in cluster 3 had no tracer condition. We also found that individuals in this high dependency cluster had a higher likelihood of presenting with both acute and chronic morbidities. They were sustaining complex medico-psychosocial problems that would require targeted healthcare interventions, such as systematic home visits and care, multidisciplinary case discussion and management, involving psychologist and social assistants. Individuals in the middle disability cluster may benefit more from health coaching strategies aiming to empower people to self-manage their health conditions, in addition to primary prevention of acute and chronic conditions. These strategies have proven useful and cost-effective in the management of chronic conditions and in averting or delaying disability [38–40]. Our findings also suggest people with health morbidities can still enjoy better cognitive, functional and social life through the transformation of their health conditions into ‘life conditions’. This may be achieved through development of Kaiser-like integrated healthcare models and health promotion programmes enabling clients to take charge of their own health to lead an acceptable and good quality life [41–44].

### Vulnerability factors associated with the population health strata

Our study also identified socio-economic risk factors of cognitive, functional and social dependency. Indeed, the odds of being in higher disability clusters were significantly higher for individuals with poor socio-economic background and empowerment, such as being a woman, elderly, rural resident and with acute or chronic morbidity. We observed that vulnerability factors such as lower socio-economic status, older age, being a female or rural resident were significantly associated with higher odds of being in higher disability clusters than cluster 1. These findings are substantiated by results from studies from both high- and LMICs [45–49]. However, education had a significant effect on disability in the bi-variable analysis but was no longer significant after adjustment for potential confounders. A multi-country study on disability–measured by WHODAS in adults aged 50 and above–found no association between education and disability in Ghana whereas a protective effect of education was reported in Russia, China, India and South Africa [46]. Post-hoc analysis in individuals aged 50 and above did not change the pattern of association in our study. This difference may be related to the heterogeneity in socio-economic structure between low-income countries like DR Congo and Ghana and middle- or high-income countries. Health status approached through disability dimensions is more common among the poorer. Thus, in low-income countries like DR Congo and Ghana, confounding by socioeconomic background may underestimate the beneficial effects of education on cognitive, functional and social disability because individuals with higher disability scores will tend to be poorer.

Though the likelihood of being in higher disability clusters was higher in rural areas in general compared to urban areas, there were clear disparities between health zones within rural areas. In fact, participants form Walungu health zone were worse off in terms of functional, cognitive and social disability compared to those living in Katana and Miti-Murhesa health zone. This difference is substantiated by the fact that Walungu zone had experienced longer and more direct effects of armed conflicts than Katana and Miti-Murhesa. It has been shown that the severity and gender dimensions of armed conflicts in Walungu has compromised family relationships and social interaction [50–52], resulting in long-lasting effects of war including post-traumatic disorders, depression, destruction of the social structure and economy of the region [53]. This protracted fragile context explains the higher burden of complex medico-psychosocial conditions observed in Walungu compared to other rural health zones and calls for rethinking healthcare programs in post-conflict regions in order to develop healthcare programs that are responsive to people’s individual healthcare needs and context.

In this study, we found no significant association between the child’s acute malnutrition status and the probability of a mother falling in higher disability clusters. We hypothesized that most severe cases of child malnutrition with a higher likelihood of impacting on the mother’s functional, cognitive and social ability were more likely taken care of as inpatients in therapeutic feeding centers rather than in the community. Future studies involving mothers of inpatient children with severe acute malnutrition and by including qualitative approaches may clarify such a link.

### Strengths and limitations

This study had some limitations. First, these findings have limited generalisability to people living with other health conditions and which are difficult to reliably identify at community level in settings where patient’s medical records are not available. Neither can our findings be generalisable to individuals severely physically or mentally impaired to the extent that they could not consent to the study or withstand the interview. However, we believe that by having extended the sampling to caregivers and randomly selected individuals in the neighborhood contributed to gaining insights in health status of individuals not presenting with the tracer conditions aforementioned and helped alleviating the effect of this potential bias. The sampling was also confined to villages close to the health centre in each health area in order to be able to assess how change in the way healthcare services are being provided at the health centre may have impacted on the health status of the population, in the framework of the research for development project on which this study draws. The sample selection was based on the assumption that people in villages far away from the health centre were more likely to seek health services from health centres in neighboring health areas, therefore would have been hard to follow up with linkage to the research for development project on which this study is drawn. in the framework of the research for development project on which this study draws. This selection might have induced a selection bias whereby individuals living in remote villages relative to the health centre may have limited access to health services, which in turn may impact on their health outcomes.

Sixty three percent of our respondents were female. This may partly be explained by the fact that the great majority of women in eastern DR Congo were housewives and more likely to be present at home when the interviewers passed by, with men moving around looking for occasional job opportunities in a region where the informal work sector or daily labour reigns. This may have resulted in a sampling bias, over representing women. We do acknowledge that such a bias might result in overestimating associations since women are more likely to score higher on WHODAS than men [54, 55]. Our results should be interpreted accordingly. However, the replication of the associations observed across different settings with heterogeneous confounding structure suggests that this potential sampling bias likely has little effect on the pattern of associations we observed.

Akin to other observational studies, our analysis is subject to residual confounding. For example, we did not have data on psychosocial factors like anxiety and depression that are shown to be associated with higher disability scores [56]. In addition, we could not directly measure the effect of family and social interactions on health status clustering. A recent systematic review stressed the link between social relationship, mental health and wellbeing in physical disability [56]. Further studies are needed to explore the extent to which these factors may influence health status clustering.

Our study also has a number of strengths. It provides a unique insight into health status clustering of individuals at community level in a post-conflict setting. Based on modern and robust cluster analysis tools, this study proposes an innovative and programmatically useful approach to measuring health status and disability of individuals using the WHODAS. Our results can guide design and implementation of appropriate healthcare programs that fit people’s needs and leverage the overall human health capital. This study also provides precise measures of associations estimates with narrow confidence intervals suggesting a sample size large enough, in a region relatively hard-to-reach and to some extent scientifically isolated.

## Conclusion

Population health stratification based on cognitive, functional social dependency at primary healthcare level may be a powerful lever for prioritization, design, implementation and scale-up of integrated care interventions with a great potential to improve quality of lives of people living in LMICs. The hierarchical health status clustering implies the necessity for a programmatic approach to the provision of healthcare services for individuals and communities in settings where resources are scarce. Our results suggest that health clustering derived from WHODAS domains scores has the potential to appropriately discriminate individuals based on the levels of health needs and increase the likelihood of appropriate healthcare service provision to all, included to those with vulnerabilities who could be easily overlooked by the usual disease-based classification of a population.

## Additional file

Additional file 1: Distribution of the summary WHODAS score and sub-group analysis. **Figure S1.** shows the distribution of the summary WHODAS score in the overall study population and by health clusters. **Table S1.** reports the morbidity factors and WHODAS domains scores of the three enrolment groups. The characteristics of informal caregivers and patients are described in **Table S2** and **Table S3** respectively. (DOCX 86 kb)

## Abbreviations

AOR: Adjusted odds ratio
COR: Crude odds ratio
DR: Democratic Republic
HT: Hypertension
ICC: Intraclass correlation coefficient
IQR: Interquartile range
LMICs: Low- and middle-income countries
SD: Standard deviation
SES: Socio-economic status
VIF: Variance inflation factor
WHO: World Health Organisation
WHODAS: World Health Organisation Assessment Schedule
