# Supplementary material for: A new look at population health through the lenses of cognitive, functional and social disability clustering in eastern DR Congo: a community-based cross-sectional study
Source: BMC Public Health. 2019 Jan 21;19:93. doi: 10.1186/s12889-019-6431-z (PMC6341676; doi:10.1186/s12889-019-6431-z)
Supplement: Supplementary file 1 — Distribution of the summary WHODAS score and sub-group analysis. Figure S1. shows the distribution of the summary WHODAS score in the overall study population and by health clusters. Table S1. reports the morbidity factors and WHODAS domains scores of the three enrolment groups. The characteristics of informal caregivers and patients are described in Table S2 and Table S3 respectively. (DOCX 86 kb) [file 12889_2019_6431_MOESM1_ESM.docx]

**A new look at population health through the lenses of cognitive, functional and social disability clustering in eastern DR Congo: a community-based cross-sectional study**

**Additional file 1**

Table of Contents

[1. Distribution of the summary WHODAS score 1](#_Toc534888062)

[2. Morbidity, functional and social disability of the study participants 2](#_Toc534888063)

[3. Sub-group analysis 3](#_Toc534888064)

# Distribution of the summary WHODAS score

Half of the study population had summary WHODAS score below 10.2 (Figure 1).

**Figure S1. Distribution of** **the summary WHODAS in the study population.**

# Morbidity, functional and social disability of the study participants

Results on the morbidity and disability of the study respondents are summarized in Table 2. The median (IQR) of the WHODAS summary score was 10.2 (1.4-25.1), unevenly distributed across the enrolment groups. Patients had unsurprisingly higher overall median score [median (IQR): 28.8(10.5-45.8)], nearly 5 times that among caregivers [6.2(0-13.3] and almost 4 times that among neighbours [7.4(0-14.7)]. For virtually all the seven WHODAS domains, patients had consistently higher scores than both caregivers and neighbours. When comparing WHODAS domains scores between caregivers and neighbours, six of the seven WHODAS domains were comparable, with neighbours scoring significantly higher in understanding and communication that caregivers (p=0.043). Three-quarter of the patients reported an acute health problem during the 30 days preceding the interview, against 23.4% of caregivers and 25.5% of neighbours (p<0.001). Up to 8.3% of the respondents had a hospital stay during the 30 days preceding the interview. Considering a 12-months recall, 15.8% of the respondents were hospitalized at least once during the past 12 months prior to the interview and patients were more likely to be hospitalized than caregivers and neighbours (p<0.001).

**Table S1. Morbidity, functional disability and morbidity of the study population by status at enrolment**

| Variable | Patients | Caregivers | Neighbours | Total | P value |
| --- | --- | --- | --- | --- | --- |
| Overall WHODAS | 28.8(10.5-45.8) | 6.2(0-13.3) | 7.4(0-14.7) | 10.2(1.4-25.1) | <0.001 |
|  |  |  |  |  |  |
| Cognition | 41.7(12.5-58.3) | 4.2(0-16.7) | 4.2 (0-20.8) | 12.5 (0-33.3) | <0.001 |
|  |  |  |  |  |  |
| Mobility | 30(5-55) | 0(0-10) | 7.5(0-30) | 5 (0-25) | <0.001 |
|  |  |  |  |  |  |
| Self-care | 0(0-25) | 0 (0-0) | 0(0-0) | 0(0-6.3) | <0.001 |
|  |  |  |  |  |  |
| Getting along with people | 20 (0-40) | 0(0-15) | 10 (0-20) | 10 (0-25) | <0.001 |
|  |  |  |  |  |  |
| Household activities | 37.5 (6.3-62.5) | 6.3(0-18.8) | 6.3 (0-25) | 12.5 (0-37.5) | <0.001 |
|  |  |  |  |  |  |
| Work or school activities | 37.5 (12.5-62.5) | 6.25 (0-18.8) | 6.25 (0-25) | 12.5 (0-37.5) | <0.001 |
|  |  |  |  |  |  |
| Social participation | 37.5 (6.3-62.5) | 6.3 (0-21.9) | 6.3 (0-21.9) | 12.5 (0-37.5) | <0.001 |
|  |  |  |  |  |  |
| Sick in the last 30 days |  |  |  |  | <0.001 |
| No | 110 (24.4) | 377 (76.6) | 345 (74.5) | 832 (59.2) |  |
| Yes | 340 (75.6) | 115 (23.4) | 118 (25.5) | 573 (40.8) |  |
|  |  |  |  |  |  |
| Hospital stay in the last 30 days |  |  |  |  | 0.003 |
| No | 370 (88.5) | 279 (92.4) | 265 (96.7) | 914 (91.7) |  |
| Yes | 48 (11.5) | 23 (7.6) | 12 (4.3)) | 83 (8.3) |  |
|  |  |  |  |  |  |
| Hospital stay in the last 12 months |  |  |  |  | <0.001 |
| No | 313 (74) | 274 (91.9) | 251 (91.6) | 838 (84.2) |  |
| Yes | 110 (26) | 24 (8.1) | 23 (8.4) | 157 (15.8) |  |

Data are n (%), median (IQR). We report row percentages.

# Sub-group analysis

**Table S2. Characteristics of caregivers**

| Variable | Cluster 1 | Cluster 2 and 3 | Total | P |
| --- | --- | --- | --- | --- |
| Zone |  |  |  | <0.001 |
| Bagira | 125 (30.1) | 5 (9.1) | 130 (36.7) |  |
| Miti and Katana | 117 (36.5) | 21 (38.2) | 138 (36.8) |  |
| Walungu | 78 (24.4) | 29 (52.7) | 107 (28.5) |  |
|  |  |  |  |  |
| Gender |  |  |  | 0.091 |
| Male | 145 (44.2) | 18 (32.1) | 163 (42.5) |  |
| Female | 183 (55.8) | 38 (14.6) | 221 (57.5) |  |
|  |  |  |  |  |
| SES |  |  |  | 0.018 |
| Least poor | 77 (23.1) | 4 (7) | 81 (20.8) |  |
| Middle | 127 (38.4) | 24 (42.1) | 151 (38.7) |  |
| Poorest | 129 (38.7) | 29 (50.9) | 158 (40.5) |  |
|  |  |  |  |  |
| Marital status |  |  |  | <0.001 |
| Never married | 76 (23.3) | 0 | 76 (20) |  |
| Married | 220 (67.5) | 48 (88.9) | 268 (70.5) |  |
| Divorced/wid/sep | 30 (9.2) | 6 (11.1) | 36 (9.5) |  |
|  |  |  |  |  |
| Age group |  |  |  | <0.001 |
| <40 | 198 (60) | 14 (25.9) | 212 (55.2) |  |
| 40-60 | 96 (29.1) | 11 (20.4) | 107 (27.9) |  |
| >60 | 36 (10.9) | 29 (53.7) | 65 (16.9) |  |
|  |  |  |  |  |
| Household size | 7 (5-9) | 5.5 (3-8) | 7 (5-9) | 0.0014 |
|  |  |  |  |  |
| Saving |  |  |  | 0.174 |
| No | 281 (85.9) | 45 (78.9) | 326 (84.9) |  |
| Yes | 46 (14.1) | 12 (21.1) | 58 (15.1) |  |
|  |  |  |  |  |
| Tribe |  |  |  | 0.048 |
| Shi | 289 (87.6) | 55 (96.5) | 43 (11.1) |  |
| Others | 41 (12.4) | 2 (3.51) | 344 (88.9) |  |
|  |  |  |  |  |
| High blood pressure |  |  |  | 0.054 |
| No | 254 (76.3) | 50 (87.7) | 304 (77.9) |  |
| Yes | 79 (23.7) | 7 (12.3) | 1. 22.1) |  |

SES: socio-economic status

**Table S3. characteristics of patients**

| Variable | Cluster 1 | Clusters 2 and 3 | Total | P value |
| --- | --- | --- | --- | --- |
| Zone |  |  |  | <0.001 |
| Bagira | 61 (43.9) | 66 (32.3) | 127 (33) |  |
| Miti Murhesa | 62 (44.6) | 65 (31.9) | 127 (37) |  |
| Walungu | 16 (11.5) | 73 (35.8) | 89 (26) |  |
|  |  |  |  |  |
| Gender |  |  |  | 0.216 |
| Male | 45 (20.8) | 52 (24.9) | 97 (27.3) |  |
| Female | 101 (69.2) | 157 (75.1) | 258 (72.7) |  |
|  |  |  |  |  |
| SES |  |  |  | 0.896 |
| Least poor | 39 (26.4) | 52 (24.8) | 91 (25.4) |  |
| Middle | 53 (35.8) | 80 (38.1) | 133 (37.2) |  |
| Poorest | 56 (37.8) | 78 (37.1) | 134 (37.4) |  |
|  |  |  |  |  |
| Marital status |  |  |  | 0.098 |
| Never married | 4 (2.8) | 2 (1) | 6 (1.7) |  |
| Married | 102 (70.3) | 130 (62.8) | 232 (65.9) |  |
| Divorced/w/s | 39 (26.9) | 75 (36.2) | 114 (32.4) |  |
|  |  |  |  |  |
| Age group |  |  |  | <0.001 |
| >40 | 37 (25.2) | 16 (7.7) | 53 (15) |  |
| 40-60 | 60 (40.8) | 72 (35) | 132 (37.4) |  |
| >60 | 50 (34) | 118 (57.3) | 168 (47.6) |  |
|  |  |  |  |  |
| Household size | 7 (5-9) | 6 (4-9) | 7 (4-9) | 0.049 |
|  |  |  |  |  |
| Saving |  |  |  | 0.209 |
| No | 109 (74.2) | 166 (79.8) | 275 (77.5) |  |
| Yes | 38 (25.8) | 42 (20.2) | 80 (22.5) |  |
|  |  |  |  |  |
| Tribe2 |  |  |  |  |
| Shi | 136 (92.5) | 192 (91) | 30 (8.4) |  |
| Others | 11 (7.5) | 19 (9) | 328 (91.6) |  |
|  |  |  |  |  |
| High blood pressure |  |  |  | 0.855 |
| No | 13§ (91.9) | 195 (92.4) | 331 (92.2) |  |
| Yes | 12 (8.1) | 16 (7.6) | 28 (7.8) |  |

SES: Socio-economic status
